# Alexithymia as a Predictor of Arousal and Affect Dysregulations when Batterers with Attention Deficit Hyperactivity Disorder Cope with Acute Stress

**DOI:** 10.3390/bs10040070

**Published:** 2020-04-01

**Authors:** Ángel Romero-Martínez, Marisol Lila, Luis Moya-Albiol

**Affiliations:** 1 Department of Psychobiology, University of Valencia, Avenida Blasco Ibañez, 21-46010 Valencia, Spain; Luis.Moya@uv.es; 2 Department of Social Psychology, University of Valencia, 46010 Valencia, Spain; Marisol.Lila@uv.es

**Keywords:** acute stress, heart rate, intimate partner violence perpetrators, pre-ejection period, trier social stress test, vagal tone

## Abstract

Empirical research has stated that Attention Deficit Hyperactivity Disorder (ADHD) might underlie intimate partner violence against women (IPVAW) perpetration. Even though there is a clear relationship between these two variables, it is still unknown how ADHD facilitates violence proneness. In this regard, psychophysiological variables such as skin conductance levels (SCL) might offer information about emotional regulation when individuals cope with stress. Furthermore, alexithymia traits might be a strong candidate in explaining the above-mentioned emotional dysregulations. Hence, we compared the SCL response to acute cognitive stress in IPVAW perpetrators with and without ADHD symptoms to that of controls (non-violent and unaffected), and we also assessed the presence of alexithymia traits and their role in emotional regulation. Our data point out that ADHD IPVAW perpetrators presented higher SCL and negative affect than controls, particularly during the recovery period. Moreover, ADHD IPVAW perpetrators showed higher self-reported alexithymia, and this variable was a good predictor of autonomic and psychological state dysregulations, even after controlling for the effects of alcohol and drug misuse. Therefore, our study reinforces the need to consider psychophysiological measurements when screening the therapeutic needs of IPVAW perpetrators, due to their relatively low cost and the significant contents of their results. Finally, we also highlight the key role of alexithymia in this violent population, which should be considered when designing cognitive intervention training coadjutant to current psychotherapies for IPVAW perpetrators.

## 1. Introduction

The World Health Organization (WHO) estimates that 35% of women worldwide have been victims of certain types of physical and/or sexual intimate partner violence at some point in their lives. This type of violence exponentially increases the victims’ risk of developing mental health disorders. It is even more worrisome that approximately 38% of assassinated women are murdered by their violent male intimate partner [1] Therefore, we need effective ways to reduce and/or prevent intimate partner violence against women (IPVAW) in heterosexual relationships. In fact, not only is it necessary to understand the effects of IPVAW on women’s health, but it is also worthwhile to acknowledge the main factors that lead specific men to mistreat or even murder their female partners. Accordingly, acquiring greater knowledge about the complex phenomenon of IPVAW will allow us to develop effective strategies to intervene and drastically reduce this problem. In addition, a successful multidisciplinary approach to IPVAW might help to prevent it and even break this cycle of violence in the long term.

Although psychologists have assessed IPVAW from cognitive-behavioral approaches in order to develop batterer intervention programs, they have paid little attention to the potential influence of biological variables [2,3]. Due to the limited results obtained until now from current interventions developed for IPVAW perpetrators, it makes sense to explore alternative points of view based on biopsychosocial models of violence [3,4], in order to design complementary and coadjutant treatments to current psychotherapeutic treatments. For example, psychotherapies complemented with pharmacological strategies, cognitive training, and/or neurorehabilitation interventions considerably increase the success of the interventions in many populations [5,6].

In recent years, empirical research has highlighted that Attention Deficit Hyperactivity Disorder (ADHD) is a risk factor for several types of IPVAW perpetration in young and adult men. In fact, unless ADHD symptoms are treated, the risk of IPVAW perpetration will remain high [7,8,9,10]. Many authors have tried to explain the facilitator role of ADHD because it usually coexists with other risk factors for violence, such as drug misuse, antisocial personality traits, previous criminal history, etc. [7,8,9,10]. Nevertheless, it should be kept in mind that although that there are clear associations between these variables, it is still unknown how ADHD symptoms condition emotional and behavioral regulation to facilitate violence proneness. 

Neuroimaging techniques offer information about brain functioning in ADHD patients, but they are still expensive. Thus, it is advantageous to include peripheral markers of brain functioning because they are cheap and can be easily adapted to different contexts. A peripheral marker of the Autonomic Nervous System (ANS), skin conductance (SC), might offer information about arousal changes in individuals. SC is a good marker of ANS functioning because the sympathetic nervous system innervates sweat glands, and so changes in emotional arousal might be easily captured through SC by registering electrodermal activation [11,12]. In this regard, the skin conductance level (SCL), which is the tonic component of SC and reflects the total electric conductivity of the skin for a short period of time lasting minutes, has been widely employed in psychology to check for ANS changes in response to stimuli [13]. 

Most individuals with ADHD tend to present autonomic arousal dysregulations in coping with acute stress [14]. Several studies concluded that children with ADHD showed a reduced SCL (hypo-arousal) during resting periods and in response to low-demand laboratory tasks, in comparison with normative individuals [15,16,17,18,19]. Unfortunately, these studies only included children. Regarding adults with ADHD, only two studies assess whether SCL in adult men with ADHD differs from that in normative individuals. These studies failed to obtain differences between ADHD and non-affected individuals [20,21]. Nonetheless, another study proposed that instead of hypo-arousal, ADHD individuals tend to present arousal dysregulations when coping with acute stress [22]; that is, they show a differential pattern of autonomic response, but not necessarily a specific hypo-arousal. These autonomic alterations in coping with stress might be a reflection of serious difficulties with introspective thinking, in other words, difficulties in identifying and communicating their feelings (alexithymia). In this regard, numerous studies have found that ADHD individuals tend to score highly on self-reports assessing alexithymia traits [23,24,25,26,27]. Therefore, it seems logical to conclude that alexithymia might be at least partly responsible for these abnormal autonomic functioning patterns in ADHD individuals.

Regarding autonomic regulation in IPVAW perpetrators, it was previously concluded that they presented a heightened SC after recovering from an acute laboratory stressor. In other words, in the period when parasympathetic activation has to increase in order to return to initial allostatic state values, IPVAW perpetrators’ sympathetic systems remain activated [28]. Later, these results were reinforced by employing other autonomic markers related to the cardiorespiratory system, leading to the same conclusion [29]. Therefore, their heightened arousal during the recovery period might be a reflection of rumination, which usually involves high levels of worry, tension, irritability, and/or hostility due to difficulties in dealing with their own inner states and coping satisfactorily with stress [30,31]. Another recent study complemented previous ones by incorporating the role of alcohol misuse in autonomic regulation in coping with stress. It was concluded that these dysregulations were more marked than in non-violent individuals because alcohol and impulsivity traits were higher [32]. Nonetheless, these studies did not pay attention to alexithymia traits and whether they play a role in explaining the differential autonomic response of IPVAW perpetrators to acute stress. We strongly believe that it would be important to consider these variables, because recent studies targeted the importance of considering alexithymia in designing intervention programs for IPVAW perpetrators [33,34].

With this in mind, in this study we first aim to determine whether IPVAW perpetrators with and without ADHD symptoms have different SCL and psychological state responses to acute laboratory stress from non-violent men. As previously demonstrated with ADHD individuals [15,16,17,18,19,20,21] and IPVAW perpetrators [28,29], ADHD IPVAW perpetrators would be expected to tend to present lower resting SCL and higher levels during the recovery period than controls. Regarding psychological changes, previous studies reported a differential psychological state stress response in IPVAW perpetrators, characterized by a higher state of anger and worse mood levels before starting a laboratory stress task [35]. Therefore, IPVAW perpetrators with ADHD traits would present higher anger and worse moods than the rest of the participants, due to their serious difficulties in dealing with their inner states [28,29,33,34,35]. Second, we explored whether alexithymia traits would explain differential patterns of response (SCL and psychological states) in all the participants. Based on previous conclusions in this field of research [33,34], we would expect higher alexithymia traits to explain autonomic (hypoarousal or hyperarousal) and psychological dysregulations (high anger and worse mood) in coping with stress. Moreover, we hypothesize that these associations will be especially significant in IPVAW perpetrators with ADHD.

## 2. Methods

### 2.1. Participants

The final sample was composed of 55 healthy male volunteers (36 IPVAW perpetrators and 19 controls). All of the IPVAW perpetrators came from the CONTEXTO psycho-educational and community-based treatment program at the University of Valencia (Spain). These men received a prison sentence of less than two years, and they did not have previous criminal records. Thus, their sentence was suspended on the condition that they attend the CONTEXTO program and complete the program in about one year [36]. 

The inclusion criteria for this study were (1) the absence of severe mental disorders (e.g., unmedicated depression, schizophrenia, mental retardation, etc.) and/or brain injuries (e.g., brain tumors, strokes, severe traumatic brain injuries etc.), (2) being drug-free during the week previous to the study, and (3) presenting good writing and speaking skills in Spanish. 

Participants were classified as ADHD depending on their performance on Conners’ Performance Test-III (CPT-III) and their average score on a validated self-report assessing impulsivity. Accordingly, the risk/suspicion of ADHD was determined when individuals presented stronger indications of inattentiveness, impulsivity, and sustained attention. In this regard, participants had to present a T-score of 0.60 (or above) on the following scales of Conners’ Continuous Performance Test-III (CPT-III): detectability, omissions, commissions, perseverations, and hit reaction time, among others [37]. Furthermore, we also considered it important for individuals to present an average score above 27 on the Spanish version [38] of the Plutchik impulsivity scale [39]. Following this procedure, IPVAW were divided into two groups according to the presence of ADHD (n = 19) or not (n = 17).

Regarding controls, we advertised for male volunteers in Valencia. In fact, we established contact by email and then screened applicants in interviews. The inclusion criteria for controls included having no physical or mental problems; having similar anthropometrical and demographic characteristics to the IPV perpetrators; and not having perpetrated severe violence, defined as assaulting a partner or other individual outside the home, or engaging in any severely violent act. To check for criminal records, we required them to provide criminal record certificates. Finally, in order to check the suitability of controls, their performance on the CPT-III was also assessed, with three participants excluded due to the indication of a strong presence of ADHD symptoms.

Before agreeing to participate in the study, all participants gave their written informed consent. This experiment was carried out in accordance with the Helsinki Declaration and approved by the University of Valencia Ethics Committee (The code is H1348835571691).

### 2.2. Procedure

Each subject participated in two sessions in the psychobiology laboratories of the University of Valencia (Spain). During the first session, all the participants were interviewed to exclude those who did not fulfil the inclusion criteria. Moreover, information was collected about anthropometric and sociodemographic variables, and variables related to violence (e.g., violence against people other than the partner). The experimental session took place in a noise-insulated room with a constant temperature of 22 ± 1°C between 4:00 and 7:00 p.m. This session lasted approximately 90 minutes, with the continuous monitoring for 40 minutes of SCL while participants were exposed to an acute laboratory stressor during this period. The stress tasks consisted of a set of neuropsychological questionnaires assessing IQ, speed processing, memory, and executive functioning that participants had to complete in front of an audience of two evaluators (a man and a woman). In order to increase the perceived socio-evaluative threat, evaluators offered feedback about their performance (e.g., “Could you be trying harder to do the tasks?”, “Is that all you can do?”, “Your colleagues scored higher on these tasks”, “We recommend that you try harder”, etc.). This study protocol has demonstrated its validity in promoting ANS changes in several populations [32,40,41]. During the SCL register, the following periods were included: resting, preparatory, stressor, and recovery (post-task). Each period lasted 5 min, except the stressor and recovery periods, which lasted 20 and 10 min, respectively. Finally, before and after the SCL register, participants completed psychological state questionnaires assessing anger and mood state.

### 2.3. Psychological State Variables

State-trait anger and its expression were measured with an adapted version [42] of the State-Trait Anger Expression Inventory-2’ (STAXI-2) [43]. This test is composed of three subscales for state of anger (feelings, verbal expression, and physical expression), all rated on a 4-point Likert-type scale (1 = “not at all” to 4 = “very much so”). A total anger score (anger state) was obtained by combining three subscales into a single variable. Cronbach’s alpha ranged from 0.67 to 0.89. 

Negative mood was evaluated through the shortened version of the Profile of Mood States (POMS) [44]. This version consists of 29 items rated on a 5-point Likert scale, where 0 is “Not at all” and 4 is “Very much”. The total score was obtained by summing the depression, anger, fatigue, and stress scores, subtracting the score for vigor, and adding a constant of 100. The Cronbach’s α was above 0.80 on all of the subscales.

### 2.4. Alexithymia Traits

Alexithymia was assessed with the Toronto Scale of 20 Elements (TAS-20) [45], validated in Spanish [46]. This test contains 20 Likert-type items rated on a 6-point scale (from 0 to 5) (i.e., “I am often confused about what emotion I am feeling”, “It is difficult for me to find the right words for my feelings”, etc.). A total score was employed as a dependent variable in this study by adding 20 items into one. The cut-off score to diagnose the presence of alexithymia is 61. Cronbach’s alpha was 0.88.

### 2.5. Alcohol Assessment

For this study, we employed the Spanish version [47] of the *Alcohol Use Disorders Identification* Test (AUDIT) [48], in order to check for the quantity and frequency of alcohol use in adults. Based on the cut-off score (>8), participants were classified as risky drinkers (i.e., with heavy and sustained alcohol consumption) or non-risky drinkers. The Cronbach’s alpha for this study was 0.78.

### 2.6. Electrophysiological Recording

We employed the Ambulatory Monitoring System (VU-AMS) to record participants’ electrodermal signals. In order to register these signals, the Biopac TSD203 was employed, combined with isotonic electrode gel (GEL101) for skin conductance, which was recorded from the medial phalanges of the index and middle or ring fingers. A yellow connector was used to record the SCL. Moreover, an infrared interface cable connected the ambulatory recording device (VU-AMS5fs) to the monitoring computer. Afterwards, data processing from electrodermal signals was computationally performed with the Data Analysis and Management Software (DAMS) (http://www.vu-ams.nl/vu-ams/software/).

### 2.7. Data Analysis

One-way ANOVAs and chi-square analyses were performed to check for significant group differences in anthropometric (age and BMI) and demographic variables (educational, income level, drug misuse, etc.).

The Shapiro-Wilk test was used to explore whether the data were normally distributed. Because SCL did not meet the assumption of normality (*p* < 0.05), we carried out nonparametric tests for statistical analysis of the results. In fact, the “time” (resting, preparatory, task, and recovery periods) effect was assessed with the Friedman test. Moreover, the interaction between “time” and “group” was calculated with the Kruskall-Wallis test.

To explore psychological state responses, after confirming the normality of the data, repeated-measures ANOVAs were performed with “time” (pre and post) as the within-subject factor and “group” as the between-subject factor. Greenhouse-Geisser corrections for degrees of freedom and Bonferroni corrections for multiple comparisons were applied where appropriate. For significant results, partial eta squared (η_p_^2^) is reported as a measure of effect size. Furthermore, in order to assess the changes between the baseline and post-task scenarios, we calculated the differences between the post-task and baseline/pre-assessment values of the psychological variables. To check for group differences in both variables, we conducted one-way ANOVAs.

The magnitudes of the stress response were estimated by the area under the curve with respect to the increase (AUC_i_) of the SCL. The trapezoid formula was employed to estimate the magnitude of the response, as we previously did with psychophysiological variables [28,29,40]. To check for group differences in AUCi, we conducted a Kruskall-Wallis test. Finally, linear regression models were constructed using the alexithymic traits (TAS-20) as the independent variable and the SCL periods (resting, preparatory, task, and recovery), the psychological state variables, and the difference between means in psychological state variables (post-task minus baseline) as the dependent variables. Furthermore, “group”, and alcohol and drug misuse were included as covariates. 

Data analyses were performed using SPSS 26.0 (SPSS IBM). All reported p-values are two-tailed, and *p* ≤ 0.05 was considered significant. Average values are expressed as mean ± SEM. 

## 3. Results 

### 3.1. Participant Characteristics and Appraisal Scores

IPVAW perpetrators did not differ from controls in age, BMI, and/or demographic variables (see Table 1). Although there were no differences between the groups in the anthropometric or demographic variables, the ADHD IPVAW group presented higher drug misuse (cocaine and marijuana) and violence against others than the rest of the groups.

### 3.2. Stress Responses

The effectiveness of the acute stressor in eliciting psychophysiological changes and group differences is explored.

**Figure 1:**
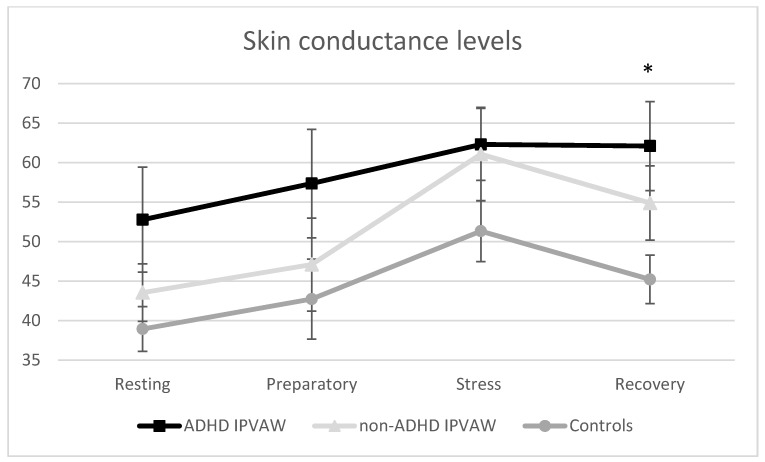
Skin conductance level changes in response to acute laboratory stress for all groups. ADHD: Attention Deficit Hyperactivity Disorder; IPVAW: Intimate Partner Violence Against Women; * *p* < 0.05.

The laboratory task employed in our study was found to be effective in eliciting SCL changes in the total sample (F Friedman (3) = 53.64, *p* < 0.001), with “time” being significant after splitting the sample into ADHD IPVAW perpetrators (F Friedman (3) = 13.23, *p* = 0.004), non-ADHD IPVAW perpetrators (F Friedman (3) = 18.53, *p* < 0.001), and controls (F Friedman (3) = 24.16, *p* < 0.001). The two experimental groups presented similar patterns of response. In fact, SCL increased from resting to preparatory time, and from then to the tasks. Afterwards, HR decreased from the task to recovery. With regard to group differences, a significant “group” effect was found in the SCL recovery period (H Kruskall-Wallis (2) = 6.67, *p* = 0.036), with ADHD IPVAW perpetrators having a higher SCL than non-ADHD IPVAW perpetrators and controls (Figure 1). Finally, there were no significant “group” differences in AUCi SCL (H Kruskall-Wallis (2) = 2.33, *p* = 0.312).

### 3.3. Psychological Responses to the Laboratory Acute Task

**Figure 2:**
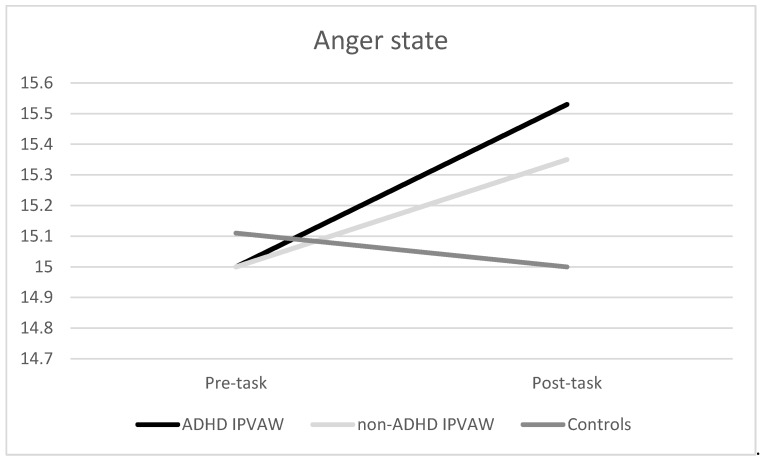
Anger state (State-Trait Anger Expression Inventory-2’ (STAXI-2)) changes before and after exposure to the acute laboratory stressor for all groups. ADHD: Attention Deficit Hyperactivity Disorder; IPVAW: Intimate Partner Violence Against Women.

For anger-state, a significant effect of “time” and the “time x group” interaction was found (F (1, 52) = 7.19, *p* = 0.010, η_p_^2^ = 0.12); F (1, 52) = 3.97, *p* = 0.025, η_p_^2^ = 0.13), respectively) (Figure 2). Although there were no significant differences between groups at baseline, both groups of IPVAW perpetrators experienced an increase in anger levels, whereas controls showed a decrease in their anger levels, after the laboratory task (F (2, 54) = 3.97, *p* = 0.025)). 

**Figure 3:**
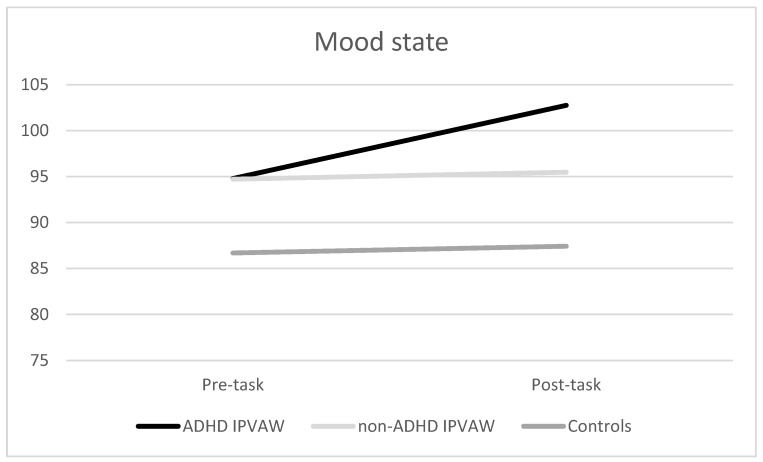
Mood state (Profile of Mood States (POMS)) changes before and after exposure to acute laboratory stressor for all groups. ADHD: Attention Deficit Hyperactivity Disorder; IPVAW: Intimate Partner Violence Against Women.

Regarding mood states, significant effects of the “time” and the “time x group” interaction were found (F (1, 52) = 5.68, *p* = 0.021, η_p_^2^ = 0.09; F (1, 52) = 3.37, *p* = 0.042, η_p_^2^ = 0.12, respectively), with ADHD-IPVAW perpetrators having worse moods at baseline and post-task (*p* < 0.05) than controls (Figure 3). Moreover, ADHD IPVAW perpetrators experienced greater changes in mood than non-ADHD IPVAW perpetrators and controls [F (2, 54) = 3.37, *p* = 0.042)]. 

### 3.4. Alexithymia Traits (TAS-20 Total Score) as a Predictor of Electrodermal and Psychological Responses to the Laboratory Acute Task, Controlling for Group and Drug Misuse (Alcohol and Other Drugs)

The TAS-20 predicted 13.3% of the SCL resting period (β = 0.386, F (1, 54) = 9.28, *p* = 0.004) and 15.1% of the variance in the SCL recovery period (β = 0.409, F (1, 54) = 10.62, *p* = 0.002). After including covariates, the TAS-20 still significantly predicted both variables (β = 0.365, t (55) = 3.05, *p* = 0.028, 95% CI = 0.04 to 0.61; and β = 0.364, t (55) = 2.34, *p* = 0.023, 95% CI = 0.05 to 0.60; respectively). Nonetheless, alexithymia traits did not significantly predict the rest of the SCL periods of SCL AUCi.

Regarding psychological variables, the TAS-20 predicted 10.8% of the variance in the anger state post-task (β = 0.353, F (1, 54) = 7.56, *p* = 0.008), 12.8% of the variance in the difference between anger state post-task minus baseline (β = 0.380, F (1, 54) = 8.93, *p* = 0.004), and 6% of the variance in the mood state post-task minus baseline (β = 0.276, F (1, 54) = 4.37, *p* = 0.041). After including covariates in step 2, the TAS-20 did not reach statistical significance. 

## 4. Discussion

Even though all of the participants showed a similar pattern of SCL changes in response to acute stress, ADHD IPVAW perpetrators had higher SCL than controls, particularly during the recovery period. Regarding the psychological response, a differential pattern of response was found. Indeed, IPVAW perpetrators (both groups) presented an increased anger state after the task and a marked worsening of mood, whereas controls showed a decrease in anger and less worsening of mood. Finally, although alexithymia traits were higher in ADHD IPVAW perpetrators than in the rest of the groups, alexithymia was a good predictor of autonomic and psychological state dysregulations in all the groups, even after controlling the effect of drug misuse. 

Contrary to our expectations in the first hypothesis, there were no differences in the general pattern of response from resting period to task in the SCL between groups. Nonetheless, we cannot conclude that ADHD IPVAW perpetrators’ SCL response to acute stress was exactly the same as that of the rest of the groups because the SCL of ADHD IPVAW perpetrators did not decrease after the task or during the recovery period, but it did in the rest of the groups (non-ADHD IPVAW perpetrators and controls). Therefore, the heightened autonomic levels during the recovery period seem to be key to reinforcing the autonomic dysregulation (not hypoarousal in this case) in adults with ADHD [22], with these differences appearing during the recovery period, but not in the resting period [28,29]. As explained above, high SCL during recovery might be indicative of a sympathetic component of ANS predominance and/or serious difficulties of the parasympathetic system to activate to regulate the allostatic state [30]. The heightened arousal might be a correlate of high levels of vigilance, hostility, and/or irritability for a long time [30], decreasing the threshold for becoming violent when faced with ambiguous stimuli (e.g., neutral facial expressions, criticism, or comments in the couple, etc.) [49]. Nevertheless, to analyze the emotional regulation of these individuals, it is necessary to assess their psychological state response to this acute stress. 

As expected, ADHD IPVAW perpetrators showed a differential psychological state response to stress in comparison with the rest of the groups, particularly controls. Indeed, they experienced a significant increase in negative affect (high anger and worsening of mood), whereas controls experienced a slight decrease in anger and worsening of mood. This increase in negative affect might be a response indicating difficulties dealing with inner states, thus affecting the way of coping satisfactorily with stress. Hence, as a second aim in our study, we proposed that the differential autonomic and psychological state responses would be explained by high alexithymia traits. In this case, our data pointed out that (1) ADHD IPVAW perpetrators presented the highest self-reported levels of alexithymia, and (2) the higher the alexithymia traits, the higher the heightened SCL response during the resting and recovery periods, as well as the increase in negative affect (high anger and worse mood). Moreover, it is important to keep in mind that we included group and drug misuse as covariates in the statistical analyses, but alexithymia still predicted autonomic and emotional dysregulations, which reinforces alexithymia’s importance in this population. Therefore, this result highlights that we need to pay attention to alexithymia when designing future batterer intervention programs [33,34]. 

Nowadays, there is no clear and specific treatment to exclusively treat alexithymia, even though it has been pointed out that it could be improved by providing training in socio-cognitive variables closely related to it [50,51,52], for example, by working with emotional decoding abilities, perspective taking, emotional verbal fluency, attentional biases toward certain stimuli, attentional deficits in processing emotional stimuli, communicative skills, theory of mind, etc. We strongly believe that in the case of IPVAW perpetrators with these needs, it would be possible to design coadjutant treatments to treat abilities and decrease alexithymia. In this regard, these socio-cognitive training programs could consist of short training sessions complementary to the main batterer intervention programs, with a 15- to 30-minute daily routine. It would be advisable to combine pen and pencil tests with computerized tasks, obviously adapted to individual capacities. We also think that these short programs would help to avoid saturating IPVAW perpetrators with therapy content, by being inserted in the middle of it. Nevertheless, it would be necessary to check their improvements with randomized control trials, as has been done until now [53].

This is the first study to point to heightened arousal of IPVAW perpetrators with ADHD symptoms in response to an acute laboratory stressor, but we recognize that the experiment has some limitations that should be considered in future studies. Nevertheless, this pilot study is part of an ongoing research effort to improve our understanding of the main variables underlying IPVAW perpetration. The research design is strong, including a control group that was matched on the main demographic characteristics and a reliable measurement of arousal changes such as SCL. First, the major limitations of the study are the small sample size and its correlational nature. For this reason, future studies should explore group differences in larger samples. Another limitation that should be highlighted is the absence of cardiorespiratory markers (e.g., heart rate, respiratory rate, etc.) and sympathetic (e.g., pre-ejection period) and parasympathetic (respiratory sinus arrhythmia) indicators, which could help to increase the value of our data. Furthermore, it would also be interesting to consider other biomarkers, such as serum cytokines, alpha amylase, testosterone, cortisol, and oxytocin to assess emotional regulation. Lastly, and most importantly, we assessed ADHD symptoms based on performance on the CPT-III and the score on a self-report, but it would be necessary to cross these data with a psychotherapist’s diagnosis of ADHD based on DSM-5 criteria. In fact, based on performance on this test, we can only have a suspicion of this disorder, and not a real diagnosis. However, the CPT-III provides a strong and reliable measurement of ADHD [37]. 

In summary, our study reveals that IPVAW perpetrators with ADHD present a probable sympathethic/parasympathethic imbalance when coping with stress that is partly explained by alexithymia. For this reason, we strongly suggest that it is necessary to consider psychophysiological measurements for screening the therapeutic needs of IPVAW perpetrators, due to their relatively low cost and the significant content of their results. In fact, these peripheral markers offer information about the central nervous system (CNS), specifically, how individuals regulate their emotions and behavior. Therefore, we can employ it to build risk profiles of IPVAW perpetrators or other violent individuals. Furthermore, our study reinforces the need to consider alexithymia as a key variable in designing cognitive training for IPVAW perpetrators. Finally, all our results shed light on the biological functioning underlying violence proneness and on the different subtypes of IPVAW perpetrator with differential therapeutic needs.

## Figures and Tables

**Table 1:** Mean ± SD of demographic characteristics and psychological variables for all groups.

		ADHD-IPVAW (n = 19)	Non-ADHD IPVAW (n = 17)	Controls (n = 19)	F ANOVA/ Chi-Square
**Age (years)**		39.53 ± 11.62	45.29 ± 12.24	43.21 ± 11.57	1.11
**BMI**		25.21 ± 2.87	25.12 ± 2.33	25.80 ± 2.60	0.38
**Nationality**					1.99
**Spanish**		79%	94%	89%	
**Other**		21%	6%	11%	
**Marital status**					0.47
**Married**		32%	35%	42%	
**Single/Divorced/Widowed**		68%	65%	58%	
**Level of education**					1.39
**Primary/lower secondary**		63%	53%	53%	
**Upper secondary**		26%	24%	32%	
**University**		11%	23%	15%	
**Employment status**					0.99
**Employed**		42%	47%	58%	
**Unemployed**		58%	53%	42%	
**TAS-20**		69.58 ± 18.08	46.17 ± 20.49	40.32 ± 20.74	11.55 ***
**Alcohol misuse (AUDIT)** **Cut-off (>8)**	**Yes, low** **Yes, high** **No**	32% 16% 52%	24% 29% 47%	32% 16% 53%	1.42
**Drug misuse**	**Yes** **No**	53% 47%	35% 65%	- 100%	13.22 ***
**Violence against others**	**Yes** **No**	68% 32%	30% 70%	-	5.46 **

*Note.* ADHD: Attention Deficit Hyperactivity Disorder; IPVAW: Intimate Partner Violence Against Women; ** *p* < 0.01; *** *p* < 0.001.
